# An Online Monitoring System for In Situ and Real-Time Analyzing of Inclusions within the Molten Metal

**DOI:** 10.3390/s24092767

**Published:** 2024-04-26

**Authors:** Yunfei Wu, Hao Yan, Jiahao Wang, Xianzhao Na, Xiaodong Wang, Jincan Zheng

**Affiliations:** 1 State Key Laboratory of Advanced Steel Processes and Products, Central Iron and Steel Research Institute, Beijing 100081, China; yfwu0018@foxmail.com (Y.W.); na_cisri@126.com (X.N.); 2 College of Materials Science and Opto-Electronic Technology, University of Chinese Academy of Sciences, Beijing 100049, China; yanhao211@mails.ucas.ac.cn (H.Y.); wangjiahao21@mails.ucas.ac.cn (J.W.)

**Keywords:** electrical sensing zone, analyzing systems, inclusions, metallic fluids

## Abstract

Traditional methods for assessing the cleanliness of liquid metal are characterized by prolonged detection times, delays, and susceptibility to variations in sampling conditions. To address these limitations, an online cleanliness-analyzing system grounded in the method of the electrical sensing zone has been developed. This system facilitates real-time, in situ, and quantitative analysis of inclusion size and amount in liquid metal. Comprising pneumatic, embedded, and host computer modules, the system supports the continuous, online evaluation of metal cleanliness across various metallurgical processes in high-temperature environments. Tests conducted with gallium liquid at 90 °C and aluminum melt at 800 °C have validated the system’s ability to precisely and quantitatively detect inclusions in molten metal in real time. The detection procedure is stable and reliable, offering immediate data feedback that effectively captures fluctuations in inclusion amount, thereby meeting the metallurgical industry’s demand for real-time analyzing and control of inclusion cleanliness in liquid metal. Additionally, the system was used to analyze inclusion size distribution during the hot-dip galvanizing process. At a zinc melt temperature of 500 °C, it achieved a detection limit of 21 μm, simultaneously providing real-time data on the size and amount distribution of inclusions. This represents a novel strategy for the online monitoring and quality control of zinc slag throughout the hot-dip galvanizing process.

## 1. Introduction

Liquid metal cleanliness serves as a crucial metric for assessing metal melt quality, with non-metallic inclusions significantly impacting this cleanliness. Inclusions disrupt metal continuity, thereby diminishing its mechanical, corrosion resistance, and fatigue properties. Aluminum inclusions primarily arise from oxidation reactions, slag on furnace walls during nucleating agent addition and melting, and erosion of refractory materials [1,2], comprising oxides, nitrides, and carbides. Al_2_O_3_, the most prevalent inclusion in aluminum alloys [3], features the largest content and widest size distribution, manifesting in aluminum alloy tissues as unevenly distributed massive inclusions, uniformly distributed finely lamellar inclusions, and diffuse micro-lamellar inclusions. Non-metallic inclusions act as common initiation sites for fatigue cracks in deformed aluminum alloys, with a significant correlation observed between the size of these inclusions and the alloy’s fatigue strength. Specifically, an increase in inclusion size corresponds to a decrease in the fatigue strength of the material [4,5,6,7]. During the hot-dip galvanizing process, zinc melt inclusions, consisting of suspended slag and surface slag, significantly degrade the steel strip’s surface quality and act as a corrosion source, thereby reducing the galvanized layer’s corrosion resistance [8]. Therefore, the evaluation of the cleanliness of the liquid metal becomes a key link in the metallurgical production process.

Current inclusion detection methods encompass the metallographic method, PoDFA (Porous Disc Filtration Analyzer), K-mold, electrical sensing zone method, and ultrasonic method [9]. The metallographic method, while simple, requires lengthy sampling, making it suitable for small-scale detection. PoDFA offers both qualitative and quantitative detection but suffers from low efficiency and high costs [10,11,12]. The K-mold method detects 60–80 μm inclusions but yields biased results for cleaner molten metals [9]. Moreover, these methods are offline and cannot provide real-time data on inclusion size distribution in metal melts. Although the ultrasonic method offers real-time monitoring, its frequency limitation gives a limit to the inclusion detection range [13]. Consequently, controlling liquid metal cleanliness necessitates developing technology for real-time, online quantitative monitoring of the full range of inclusion sizes and distributions.

In the 1950s, Coulter introduced the electrical sensing zone (ESZ) method for detecting particulates in liquids via electrical resistance changes [14]. In 1985, McGill University applied this principle to metallurgy, coining it LiMCA [1]. ABB commercialized LiMCA for metallurgical industry cleanliness evaluation, leading to continuous improvements and the development of LiMCA I, II, CM, and III [15,16]. The application of ESZ in liquid metal presents more significant challenges compared to particle measurement in salt solutions. The resistivity of liquid metal is considerably lower than that of salt solutions, necessitating a higher current to enhance the signal-to-noise ratio. Furthermore, the viscosity and surface tension of liquid metal, which influence its flow through the electrical sensing zone, vary with temperature. Therefore, it is essential to apply appropriate air pressure to ensure the smooth passage of liquid metal through the ESZ. Despite its success in various industries, the electrical sensing zone principle’s application to zinc melt inclusions remains underexplored, warranting further research on its feasibility and monitoring equipment development.

This paper aims to develop a system based on the method of ESZ for real-time analyzing of the size and distribution of non-metallic inclusions in metallic liquids. The system adopts a modular design, consisting of a pneumatic module, an embedded module, and a host computer module, to carry out real-time online measurement studies of metallic fluids in different metallurgical processes.

## 2. Electrical Sensing Zone Principle and Detection Device

### 2.1. Principle of Electrical Sensing Zone Measurement

**Figure 1:**
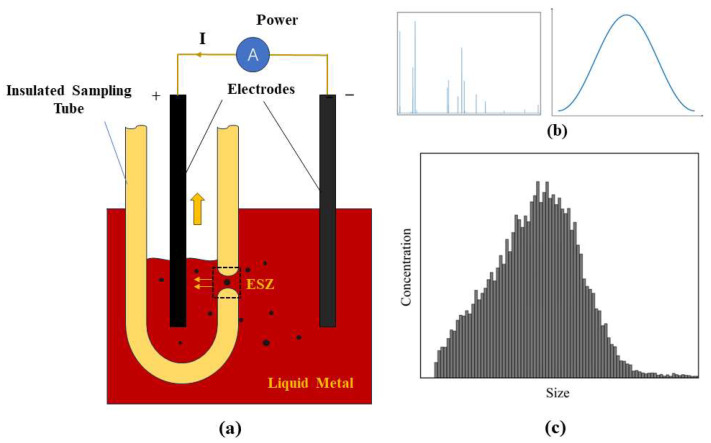
(**a**) Schematic diagram of the method of the electrical sensing zone; (**b**) pulse signal and its details; (**c**) size and concentration distribution of inclusions.

The electrical sensing zone method, depicted in Figure 1a, involves a sensor comprising an insulated sampling tube equipped with small perforations along its sidewall. Inside and outside this sampling tube, positive and negative electrodes are positioned to maintain a constant current flow. During measurements, a negative pressure is applied to the sampling tube, drawing liquid metal and its inclusions through the perforations, which alters the resistance within these openings. The presence of a constant current amplifies this resistance change, leading to a potential variation between the electrodes. This potential shift occurs exclusively within the perforations, designating this region as the ESZ.

The voltage pulse generated as a particle traverses the ESZ encapsulates significant information about the particle: the pulse’s amplitude indicates the particle’s diameter, the width reflects the particle’s velocity through the ESZ, and the pulse count denotes the number of particles passing through the ESZ within the liquid metal. This method offers a principle-based advantage and boasts high measurement precision. Inclusion concentration is commonly utilized in the industry as a criterion for assessing the cleanliness of liquid metal, defined by the number of inclusions per unit mass. This metric, expressed as thousands of inclusions per kilogram of liquid metal (k/kg), facilitates a precise evaluation. During the measurement process, the height of the liquid metal drawn into the insulated sampling tube is determined by a liquid level detection sensor within the measurement probe. These data, combined with the tube’s diameter and the liquid metal’s density, allow for the calculation of the drawn liquid metal’s mass. The inclusion concentration is then derived by dividing the detected number of inclusions by this mass. To obtain a statistical distribution of inclusions in liquid metal, the number of inclusions and the mass of extracted liquid metal over a specific period are tallied, enabling the calculation of inclusion concentration and the creation of a corresponding histogram, as depicted in Figure 1b,c. The correlation between the voltage pulse amplitude and particle size is governed by the following Equation (1):

(1) $$∆R=\frac{4\rho_{e}d^{3}}{\pi D^{4}}$$

where: $\rho_{e}$ is the resistivity of the metallic liquid; d is the nominal diameter of the particulate matter; D is the diameter of the side pore.

### 2.2. Detection Device

**Figure 2:**
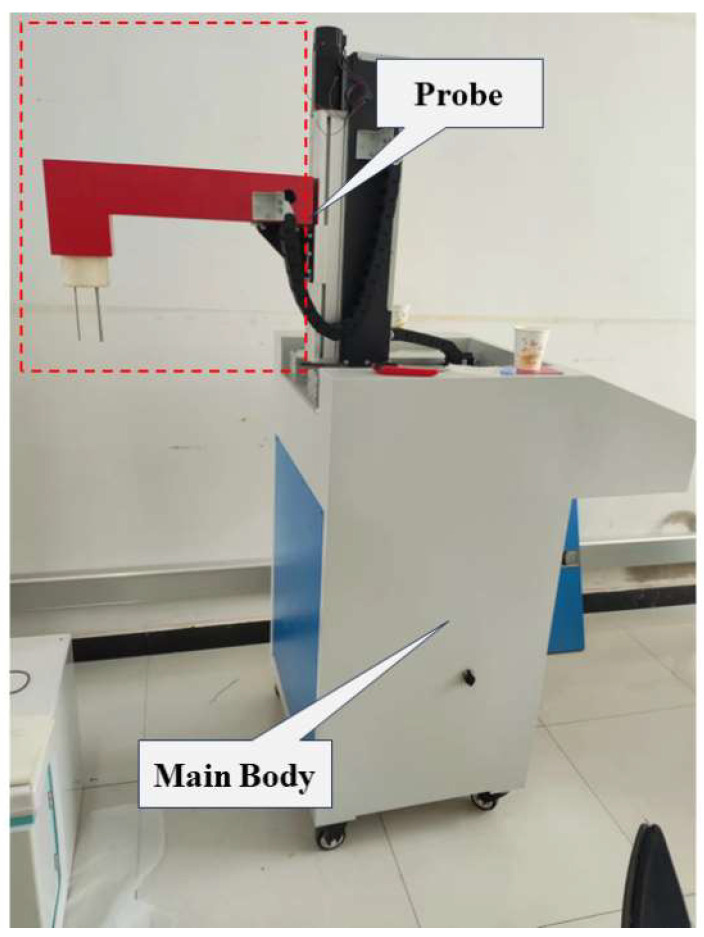
Structural appearance of the detection device.

The cleanliness online analyzing system, equipped with a detection device, as illustrated in Figure 2, comprises two main components: the main body and the probe. The main body encompasses pneumatic modules, power modules, cooling modules, and collection circuit boards, among others, while the probe incorporates the sampling tube, sampling probe, ESZ electrodes, and protective device. The probe’s movement, both forward and backward as well as upward and downward, is controlled by two stepper motors. This configuration enables the detection device to accurately measure inclusions at various locations and depths within the liquid metal.

## 3. Liquid Metal Cleanliness Online Analyzing System

**Figure 3:**
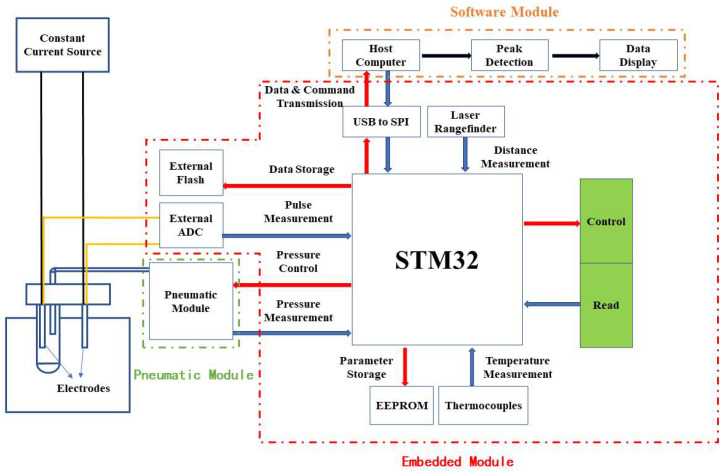
Instrument module architecture diagram (red arrow indicates data transmission from the MCU to the peripheral, blue arrow indicates data transmission from the peripheral to the MCU, black arrow represents data processing by host computer software, black thin line is the power line, yellow thin line is the signal line).

The liquid metal cleanliness online analyzing system features a modular design, with each module working in tandem to facilitate the real-time analyzing of inclusions, as illustrated in Figure 3. The pneumatic module’s function is to deliver stable air pressure, which is crucial for the consistent operation of the liquid metal’s circulation pump. An air pressure sensor continuously monitors the air pressure within the sampling tube, converting these data into digital signals via an onboard analog-to-digital converter (ADC) for transmission to the microcontroller unit (MCU). The embedded module’s responsibilities encompass signal acquisition, quantization, and transmission, alongside mechanical control. It processes voltage signals from the electrodes, amplifying and filtering them before an external ADC digitizes these signals for the MCU. This MCU then compiles voltage, laser distance sensor, and thermocouple signals into data packets for transmission to the host computer or storage in external flash memory. The software module offers an interactive interface for operators, handling raw signal processing, user command execution, and data display. Host computer software further refines voltage pulse signals through peak detection, digital filtering, and particle size conversion, displaying results on the interface. It also allows for the adjustment of other module parameters, such as output air pressure and signal gain.

### 3.1. Pneumatic Module

**Figure 4:**
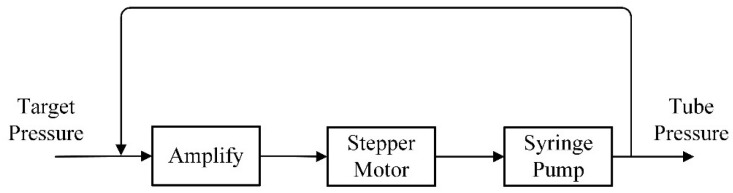
Schematic diagram of the principle of the regulating system.

The pneumatic module is critical for ensuring the stability of the liquid metal’s circulation and pumping process. In this study, the pneumatic module comprises a syringe pump, a pressure stabilization module, and a pneumatic pressure sensor. The syringe pump, central to the pneumatic module’s functionality, allows for the adjustment of the output air pressure through the controlled movement of its piston. This pressure is continuously monitored by the pneumatic pressure sensor. Due to the instability of air pressure changes caused by the movement of the syringe pump piston, a pressure stabilization module with negative feedback regulation is introduced into the air circuit design to stabilize pressure fluctuations. The operation principle of this pressure stabilization module is illustrated in Figure 4. Operators can set a target air pressure using the host computer software. The MCU compares the target air pressure with the actual output from the pneumatic module, adjusting it based on a predefined function. This adjustment is achieved by controlling a stepper motor that operates the syringe pump, thereby modifying the module’s output air pressure. The inclusion of the pressure stabilization module allows for the minimization of air pressure fluctuations around the target value, thus ensuring the liquid metal’s stable and continuous pumping.

### 3.2. Embedded Module

The embedded module constitutes the pivotal element of the analyzing system, tasked with capturing and quantifying inclusion pulse signals, retrieving instrument status sensor data, and executing mechanical controls. This module’s hardware centers around the STM32F429, a product of STMicroelectronics, characterized by its main operating frequency of up to 180 MHz and an extensive array of 21 communication interfaces. These include eight Universal Synchronous/Asynchronous Receiver/Transmitter (USART) serial ports, three Inter-Integrated Circuit (I^2^C) interfaces, and six Serial Peripheral Interface (SPI) interfaces, among others. Moreover, the MCU supports two distinct 12-bit analog-to-digital (DAC) converters and facilitates 16-channel direct memory access (DMA). The STM32F429 engages in communication with peripherals—such as external ADCs, memory modules, and data transmission units—via SPI, I^2^C, and USART protocols. It also oversees the operation of mechanical devices, like solenoid valves and stepper motors, through General Purpose Input/Output (GPIO) pins. On the software front, the embedded system employs the STM32 standard library and the Hardware Abstraction Layer (HAL) library for managing digital signal reception and transmission, as well as controlling stepper motors and solenoid valves.

#### 3.2.1. Acquisition and Quantification of the Inclusion Pulse Signal

The capture and quantization of inclusion pulse signals are crucial functions of the embedded module and are essential for determining the measurement sensitivity of the online cleanliness-analyzing system. The voltage signal, transmitted from the positive and negative electrodes to the circuit board via the signal line, undergoes filtration through a capacitor and an active power filter. The capacitor effectively removes the DC component and low-frequency noise, significantly minimizing signal baseline drift. The active filter circuit employs the ADA4004 operational amplifier from analog devices for signal amplification and filtering. The gain and passband characteristics of the signal are determined by the resistance and capacitance values within the feedback loop. In this system’s circuit architecture, the feedback loop of the active filter is connected not directly to resistors and capacitors, but through the 74HC4051 chip. This eight-channel multiplexer, developed by Texas Instruments (Dallas, TX, USA), includes three digital selectors and eight independent input/output terminals. Within the embedded module, these terminals enable access to resistors and capacitors with varying parameter values, allowing for the selection of specific resistance and capacitance values for the feedback loop via the three digital selectors. Instead of using fixed parameters, a multiplexer facilitates the integration of resistors and capacitors of various values. This approach enables real-time adjustments to the active filter circuit’s parameters, significantly enhancing system stability across diverse testing environments.

Since the MCU processes only digital signals, the analog signals, once amplified and filtered, must be digitized by the ADC before they can be interpreted by the MCU. The ADC’s conversion accuracy and sampling frequency are pivotal in minimizing signal distortion—the higher these metrics, the lesser the distortion. This system employs the LTC2500-32 chip as an external ADC, featuring conversion accuracy of up to 32 bits and a maximum sampling frequency of 256 kHz. The LTC2500-32 is equipped with two data transmission ports, labeled A and B, which facilitate the transfer of the converted digital signals to the MCU via SPI protocol. Port A is designated for transmitting digital signals with 32-bit precision, processed by an internal filter, whereas Port B is reserved for conveying unprocessed digital signals with 24-bit precision. To ensure precise conversion of digital signals back to analog form, the system preferentially utilizes Port A, given its superior conversion accuracy. Given that a typical pulse signal’s transient duration ranges from 0.1 ms to 1 ms, with an estimated frequency spectrum of 1 kHz to 10 kHz, the sampling frequency, according to Nyquist’s theorem, should be at least twice the signal’s highest frequency, thus exceeding 20 kHz. However, a higher sampling frequency, while improving analog signal fidelity, may decrease conversion accuracy, escalate data storage costs, and introduce high-frequency noise. Consequently, this system’s ADC sampling frequency is set to 62.5 kHz to balance these factors. The quantized noise signal-to-noise ratio is an important index to measure the quality of analog-to-digital conversion [17], and is determined using the following equation:

(2) $${\mathrm{SNR}}_{Q}=6.02B-20\mathrm{lg}\left(\frac{X_{m}}{\sigma_{x}}\right)+10.8$$

where B represents the number of bits of sampling accuracy, $X_{m}$ is the reference voltage, and $\sigma_{x}$ is the root mean square value of the pulse signal. The reference voltage and sampling accuracy both influence the quantization noise ratio. A higher sampling accuracy leads to a lower quantization noise ratio, while an excessively large reference voltage increases quantization noise, and an excessively small reference voltage can distort large signal amplitudes. Consequently, the closer the reference voltage is to the maximum value of the analog signal, the better. In this project, the maximum peak voltage of the pulse signal is approximately 60 mV, and ADC reference voltage is 3.3 V. With a signal amplification factor of 50, the maximum value of the amplified analog signal is 3 V. Assuming the pulse signal can be approximated as a sinusoidal waveform, the quantization signal-to-noise ratio of the analog-to-digital conversion can be calculated as 199.6 dB. By selecting a high-precision ADC and setting appropriate parameters, the quantized digital signal can achieve a high-quantization SNR and accurately represent the analog signal.

#### 3.2.2. Other Sensor Signal Acquisition

Barometric pressure sensors, thermocouples, and other signals play crucial roles in coordinating and facilitating cooperation between modules to ensure the continuous and stable operation of the system. The Sinomeasure pressure sensor, model SN-P310, features a response time under 25 milliseconds (ms) and is capable of measuring pressures ranging from −100 to 100 kilopascals (kPa), with an output voltage span of 0 to 10 volts (V). The analog signal from the barometric pressure sensor is transmitted to the MCU, converted into a digital signal by the onboard ADC, and temporarily stored in registers. Simultaneously, to reduce the load on the MCU, DMA is employed to transfer data directly from the registers into memory. During high-temperature testing, the sampling tube can often crack due to insufficient preheating. Utilizing thermocouples to monitor the temperature inside the sampling tube in real time enables sufficient preheating before high-temperature tests, thereby avoiding cracking. The system employs a K-type thermocouple manufactured by Chenyi Company (Hangzhou, China). Its signals are transmitted to the MCU through a thermocouple digital converter, which incorporates an ADC, features cold-end compensation and detection capabilities, and can monitor ambient temperatures ranging from 0 °C to 1024 °C with a resolution of 0.25 °C. The combination of thermocouples and digital converters facilitates high-precision monitoring of the temperature within the sampling tube, significantly reducing the possibility of cracking. Furthermore, laser distance sensors determine the height of the measuring probe and the liquid metal surface, preventing the probe height from becoming too low and leading to high-temperature damage to the internal components. The digital signal from the laser distance sensor can be transmitted directly to the MCU through the serial port.

### 3.3. Software Module

**Figure 5:**
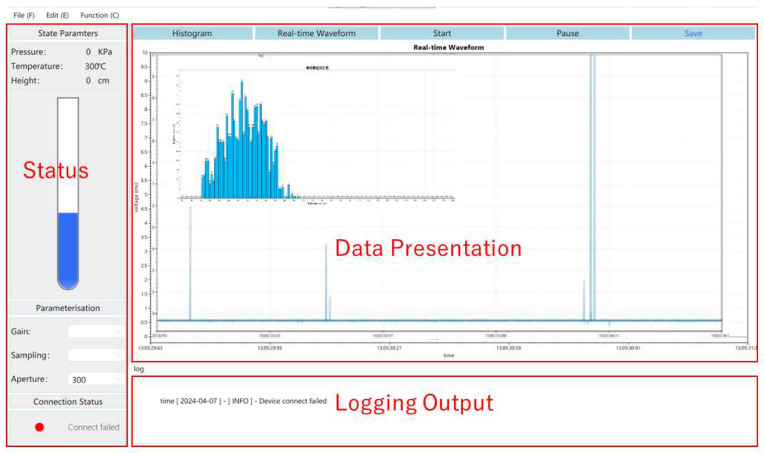
Host computer software interface and its function area division.

The host computer software serves as the interface between the user and the system, enabling users to manipulate the host computer to view the system status and test data in real time and set test parameters. The software is developed in C# using the Windows Presentation Foundation (WPF) framework within Microsoft Visual Studio, adhering to a front-end separation architecture that ensures high stability and maintainability. As illustrated in Figure 5, the host computer interface of this system can be divided into three functional areas: data display, logging output, and status. The data display area presents various forms of dynamic data to the user and updates them in real time. The log area has the capability to record user operations and system parameters, while the status area is primarily used for displaying and setting system status parameters.

#### 3.3.1. Data Transmission

**Figure 6:**
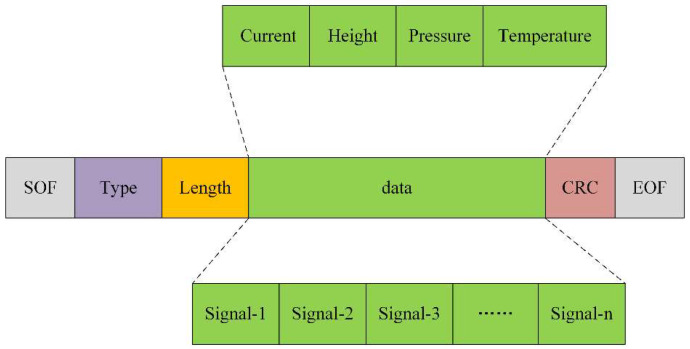
Schematic diagram of communication protocol between MCU and host computer.

The data exchange between the host computer and the MCU involves complex data types and a substantial amount of data. Direct transmission could cause data confusion, misalignment, or even loss. Consequently, the communication between the host computer and the MCU necessitates a comprehensive communication protocol to ensure the accuracy and integrity of data transmission. In this system, we employ a set of customized communication protocols, and the structure of the data frame is illustrated in Figure 6. It comprises a header, a tail, and data in between. The frame header includes the start-of-frame flag, type parameter, and data length, with the type parameter specifying the data type. This analyzing system has two primary data types: one includes state parameters, such as excitation current, probe height, sampling tube air pressure, and temperature; the other consists of voltage signal data at the two electrode ends. Since the ADC conversion precision is 32 bits, the size of each voltage data point is 4 bytes. The data length records the number of data points to be transmitted, and its size is 1 byte, limiting the data portion of the data frame to a maximum of 1020 bytes. The frame header contains the basic information of the transmitted data, and the host computer software selects different functions to parse the data frame according to the header, thus avoiding confusion between different data types. The frame tail consists of a data check code and an end-of-frame flag. The data check can effectively ensure the accuracy of data transmission and prevent data misplacement, while the end-of-frame flag represents the termination of a data frame.

**Figure 7:**
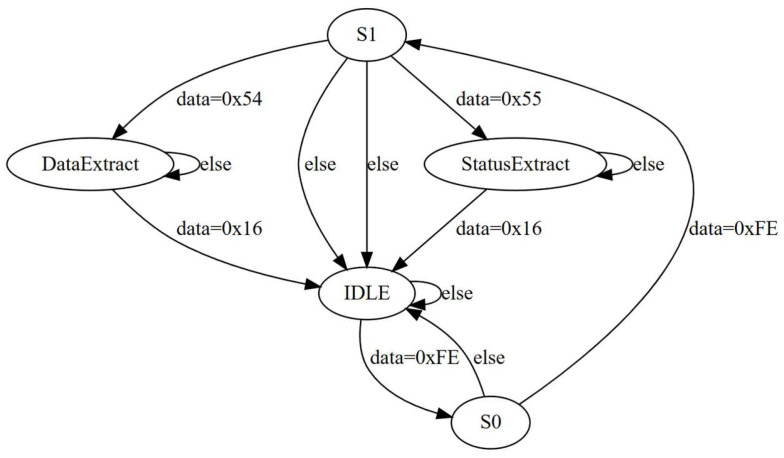
State transfer diagram for data frame parsing.

The host computer utilizes a finite state machine to parse the data packets from the MCU and extract the data portion [18,19]. In Figure 7, the parsing process adheres to a specific state transition logic, commencing from the idle state. The transition to the appropriate data extraction state occurs only if the starting frame field in the frame header contains two 0 × FE bytes, determined by the data type code. The transitions triggered by the starting frame flag’s bytes are designated as S0 and S1, respectively. Upon encountering a data type code of 0 × 54, the state machine transitions into the pulse signal extraction state, whereas a code of 0 × 55 leads to the instrument parameter extraction state. Following the completion of these processes, the state machine reverts to the idle state if the end-of-frame flag is 0 × 16. Utilizing a finite state machine model, the host computer analyzes the data packets sent by MCU, orchestrating transitions between different states based on the data frame’s type parameters. This facilitates the effective extraction, analysis, and processing of various signals. This analyzing system generates a substantial amount of data during the measurement process, with rapid data transmission speed and complex data types. The data transmission process is susceptible to errors due to various interferences. By adopting customized communication protocols for data transmission and employing a finite state machine to parse the data frames, the analyzing system significantly reduces the possibility of confusion, misalignment, and data loss during data transmission and parsing, ensuring real-time, intact, and accurate data transmission.

#### 3.3.2. Peak Detection and Data Display

**Figure 8:**
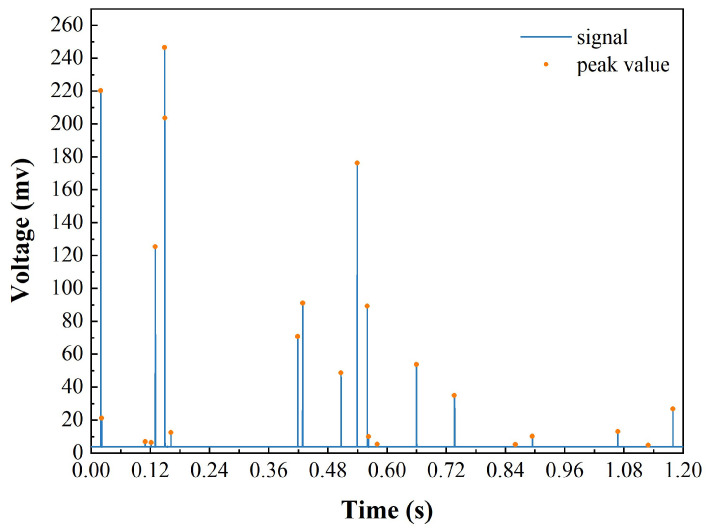
Pulse timing diagram and signal peak detection schematic.

The data transmission speed between the host computer and the MCU is rapid, and the substantial amount of data received within a short period may be too overwhelming to process promptly, potentially leading to data loss or even software interface lag. To mitigate this issue, the host computer in this inclusion analyzing system allocates a larger memory segment as a ring queue buffer. This buffer is sufficiently spacious to temporarily store a substantial amount of data, which are updated in real-time at the same rate as the ADC sampling rate. The buffer data are processed and displayed in the data display area of the host computer interface, where the data are presented in the form of real-time waveforms and histograms of the particle size distribution. The data source for the real-time waveform is termed the real-time frame, which is regularly updated by extracting data from the buffer pool and, subsequently, displayed in the data display area. It is noteworthy that the update rate of the real-time frame data must be synchronized with the update rate of the buffer pool, and the amount of data updated each time must be smaller than the initialization space of the real-time frame; otherwise, data will become unsynchronized or potentially lost. The data for the particle size distribution histogram are derived from the inclusion distribution data after pulse extraction, peak detection, and particle size conversion. The host computer extracts the voltage pulse peaks from the raw data, converts them into inclusion sizes, counts the frequency of inclusions of different sizes, and ultimately displays them as a particle size distribution histogram in the host computer interface. Unlike the analog signal processing method employed by Doutre [1], which utilizes pulse sampling for pulse extraction, this system employs a peak detection algorithm within the host computer software for pulse extraction. The algorithm operates on a principle of comparing the voltage signal at each point with those of its preceding and subsequent n sampling points. A point’s voltage is identified as a pulse peak if it surpasses the voltages of its adjacent two n points. This peak is then evaluated against a noise threshold to discern the pulse amplitude of the inclusion signal. The parameter n, crucial to the algorithm, is contingent upon the pulse signal’s transient time and the sampling rate. An increased concentration of inclusions elevates the quantity of pulse peaks to be assessed against the noise threshold, potentially impacting the peak detection algorithm’s efficiency. The pulse extraction results of this method are shown in Figure 8. The yellow dots in the figure represent the identified pulse peaks; as illustrated in Figure 8, all pulse peaks can be correctly identified and extracted, and each inclusion generates a distinct pulse signal, implying that a greater number of pulse signals indicates a higher number of inclusions. Correspondingly, the magnitude of a pulse signal is directly related to the size of the inclusion it represents. Combining a certain number of pulses with the particle size conversion formula yields statistical results for the particle size distribution. The data frame parsing and data post-processing in the host computer are performed in real time, rendering the data displayed in the host computer in real time as well. The real-time waveform graph is updated 10 times per second, and the particle size distribution histogram is updated three times per second. In this manner, real-time monitoring data from the system’s back-end can be displayed in the front-end software interface in real-time, which can serve as the basis for cleanliness quantification and process analysis.

## 4. Monitoring and Analysis of Cleanliness in Metal Melts

### 4.1. Monitoring of Inclusions in Gallium Liquids

In this paper, a cleanliness online analyzing system was employed to measure the distribution of inclusions in gallium liquid metal to verify the monitoring capability of the system. The experiment was divided into two groups:

(1) Direct measurement of the size distribution of endogenous inclusions in an untreated gallium liquid.
(2) Measurement of the inclusion size distribution in the gallium solution by adding 0.4% alumina particles with a 30 μm particle size to the gallium solution.

The sampling tube used in this experiment had a pore size of 300 μm, and the gallium solution was heated to 90 °C in a WB100-1 thermostatic water bath with constant stirring to prevent particle flotation or sedimentation. The monitoring capability of the system was evaluated by observing the two sets of measurement signals and comparing the measurement data.

#### 4.1.1. Relationship between Pulse Amplitude and Pulse Width

**Figure 9:**
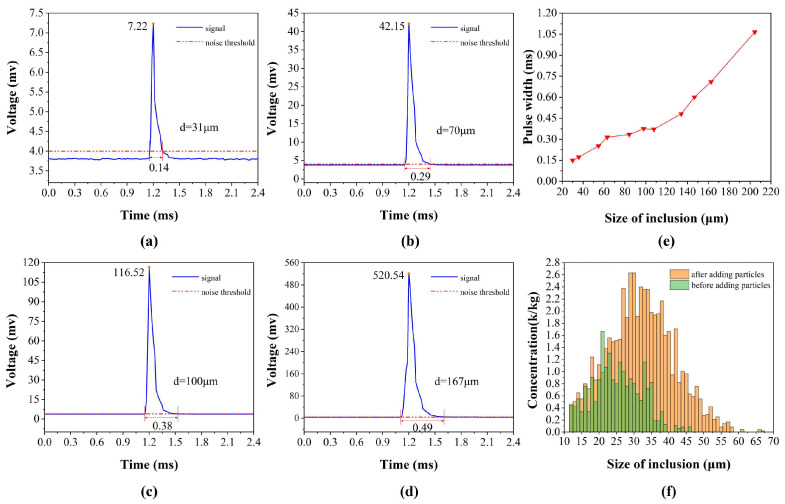
(**a**) The 31 μm inclusions pulse peak and width; (**b**) 70 μm inclusions pulse peak and width; (**c**) 100 μm inclusions pulse peak and width; (**d**) 167 μm inclusions pulse peak and width; (**e**) the trend of the change of the pulse width with the particle size; (**f**) before and after the addition of particles, the size and concentration distribution of particles in the gallium solution.

Pulse amplitude and pulse width are pivotal attributes of the pulse signal, with the former indicating the size of inclusion particles and the latter reflecting the velocity of inclusions traversing the ESZ, primarily governed by the flow rate of liquid metal through the ESZ. With constant negative pressure in the sampling tube, a reduction in the ESZ aperture size results in an increased liquid metal flow rate and a consequent decrease in pulse width. Nonetheless, Guthrie [20] demonstrated through numerical simulations that these parameters are also influenced by the density and size of the inclusions: for particles denser than the liquid metal, both the amplitude and width of the pulse signal exhibit a positive correlation, whereas for particles less dense, the correlation is inverse. Importantly, pulse width remains unaffected by inclusion concentration. Figure 9a–d illustrate the details of the pulse signals extracted from the real-time waveforms with varying pulse amplitudes, including the pulse amplitude and pulse width at a noise threshold of 4 mV. It is noteworthy that the shapes of the measured pulse signals do not match the theoretical signals, partly due to the non-spherical shapes of inclusions in the gallium liquid and partly due to the variation of the pulse waveforms caused by the front-end signal processing. It can be observed that the system can measure inclusions with a minimum particle size of 12 μm. The width of the pulse with an amplitude of 7.22 mV is only 0.14 ms, while the width of the pulse with an amplitude of 52.54 mV is 0.49 ms, indicating that the pulse width of the signal with a larger amplitude is also larger, i.e., the larger the particle size, the slower the speed of the particles passing through the ESZ. To further investigate the relationship between signal amplitude and pulse width, the researchers counted the pulse widths corresponding to pulse signals ranging from 20 μm to 106 μm. The results, shown in Figure 9e, demonstrate a positive correlation between pulse width and signal amplitude: the pulse width increases with increasing signal amplitude. The researchers also found that not all pulse amplitudes and widths are positively correlated when counting the signal pulse widths, and a small portion of the signals with larger pulse amplitudes have smaller pulse widths, due to the presence of a portion of particles in the gallium liquid whose densities are smaller than that of the liquid metal. The experimental results corroborate the numerical simulation work [20] of other scholars and illustrate the reliability of the system tested.

#### 4.1.2. Sensitivity of Measurement Results to Changes in Inclusion Concentration

Figure 9f depicts the concentration of differently sized particles in the gallium solution before and after the addition of alumina particles. Prior to the addition of alumina particles, the maximum size of inclusions in the gallium solution was 46 μm and the minimum was 12 μm, with the largest concentration of particulate matter of size 21 μm. After the addition of 30 μm alumina particles, the concentration of particles above 25 μm increased, the size of the particles with the largest concentration became 28–29 μm, and the largest size of particles measured by the system reached 67 μm. It can be seen that after the addition of alumina particles, the concentration of particles around 30 μm in size increased significantly, and large-sized particles above 50 μm appeared. These large particles are partly added to the liquid metal when alumina particles are added, and partly formed due to the aggregation of small-sized particulate matter. The changes in the size and distribution of inclusions in the gallium melt before and after the addition of the alumina particles indicate that the system is capable of measuring changes in the concentration of inclusions in the metal melt and reflecting them in the measurement results.

### 4.2. Measurement Experiment of Inclusions in Aluminum Melt

To verify the reliability of the liquid metal cleanliness online analyzing system under high-temperature conditions, the inclusions in the aluminum melt were measured using the detection device equipped with the online analyzing system. The test samples were taken from the aluminum melt in the melting process of a recycled aluminum foundry in Shandong Province, using a sampling tube with a pore size of 500 μm. The samples were placed into a muffle furnace, and the holding temperature was set at 800 °C. After heating, the system was used to measure the inclusions in the aluminum melt. Following the heating process, the samples were measured continuously for one hour using the system, and a set of inclusion distribution data was recorded every four minutes.

**Figure 10:**
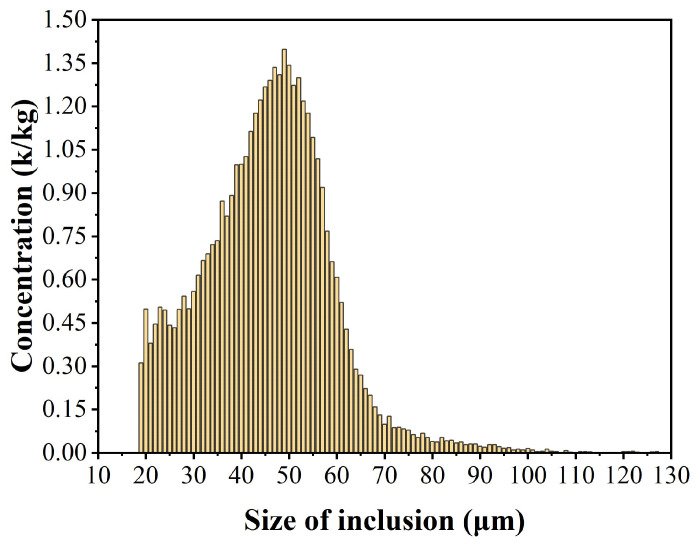
Measurement results of online inclusion analyzing system in an aluminum melt.

The 15 sets of data from the melting process samples were homogenized to obtain a histogram of the inclusion size distribution, as shown in Figure 10. The largest inclusions in the melting process samples could reach 150 μm, and the smallest inclusions that could be measured were 19 μm, with the largest concentration of inclusions having a particle size of 49 μm, which was 1.39 k/kg. According to the results in the authors’ other work [20], in the samples from the holding process and filtration process, the inclusions with the largest concentrations were 39 μm and 21 μm in size and 1.03 k/kg and 0.74 k/kg in concentration, respectively. The maximum size of inclusions measured in the holding process sample was 150 μm, while the maximum size of inclusions in the filtered samples was 72 μm. After the measurement and analysis of the size of inclusions and their concentrations in the melting process samples in the aluminum casting process, and combining the data from the authors’ other works on the holding process and filtration process data, it was found that the size and concentration of inclusions in the aluminum melt during the melting, holding, and filtration processes are gradually reduced. These results are consistent with the actual situation in the industry, which further demonstrates that the system can achieve the monitoring and evaluation of inclusions in the aluminum melt in industrial applications.

### 4.3. Measurement Experiment of Inclusions in Zinc Melt

To verify the ability of the system to detect inclusions in the zinc melt, the inclusions in the zinc melt were monitored using a testing device equipped with a cleanliness online analyzing system. The samples were taken from the zinc melt in the hot-dip galvanizing process of a steel mill in Shanghai. The sampling tube used for this test had a pore size of 500 μm. The crucible containing the zinc melt sample was heated to 500 °C in a muffle furnace and held for a sufficient period for the test. Two sets of indicators were defined for this test: N20 is the number of inclusions above 20 μm per kilogram of zinc liquid (in thousands), in units of quantity per thousand per kilogram (k/kg); and C20 is the number of inclusions between 20 μm and 30 μm per kilogram of zinc liquid (in thousands), in units of quantity per thousand per kilogram (k/kg).

**Figure 11:**
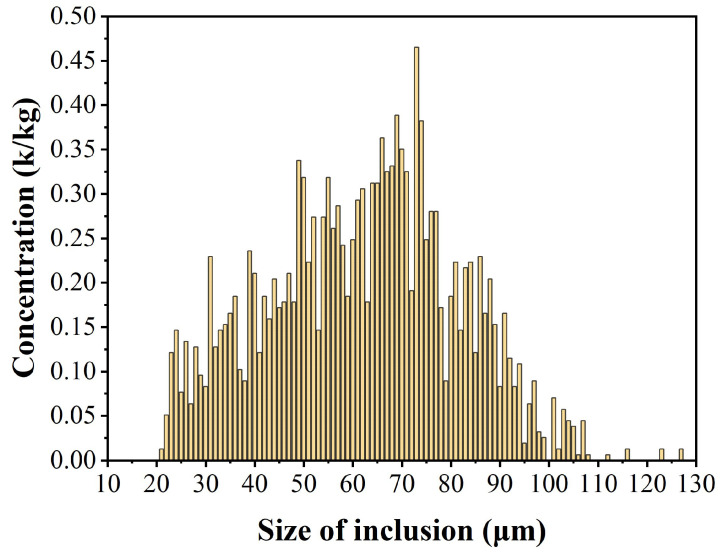
Measurements in zinc melts by an online inclusions analyzing system.

**Figure 12:**
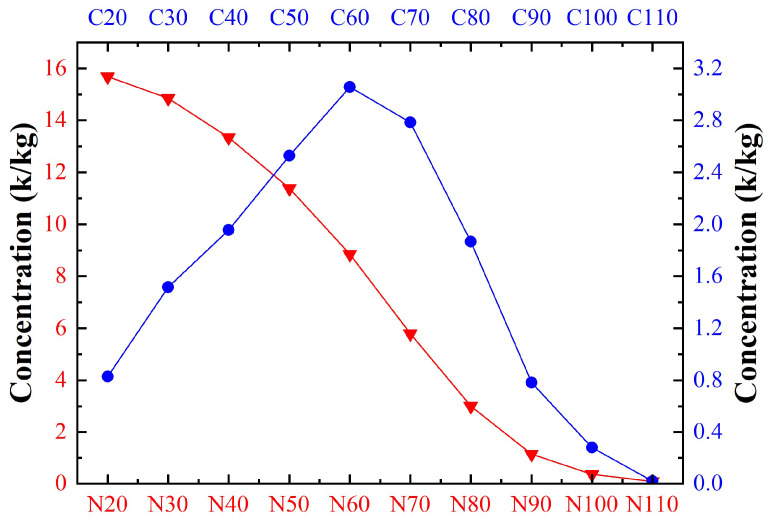
Measurements of N20~N110 and C20~C110 in zinc melts.

As shown in Figure 11, the size of the minimum inclusion measured by the analyzing system in the zinc melt was 21 μm, and the maximum inclusion size was 127 μm. Compared with the distribution of inclusions in the aluminum alloy melting process, there were more large-sized inclusions in the zinc melt, and the average concentration of inclusions with sizes between 65 μm and 75 μm was larger, reaching 0.37 k/kg. The average concentrations of inclusions between 21 μm and 64 μm and between 76 μm and 110 μm were higher than those of the aluminum alloy melt, at 0.19 k/kg and 0.10 k/kg, respectively, while inclusions above 110 μm were almost absent. Figure 12 shows the data of N20 to N110 and C20 to C110 in the zinc melt. The concentration of inclusions in N20 to N110 shows a decreasing trend, and the decreasing trends of N20 to N60 and N80 to N110 are slower, while the decreasing trend of N60 to N80 is steeper, indicating that the concentration of inclusions between 60 μm and 80 μm is higher. The values of C50, C60, and C70 are the largest in the zinc melt, with concentrations of 2.53 k/kg, 3.06 k/kg, and 2.78 k/kg, respectively, while the data for other sizes are below 2.00 k/kg, indicating that most of the inclusions in the zinc melt are distributed in the range of 60 μm to 80 μm, with a concentration of 53.37%.

The inclusion analyzing system is capable of real-time detection of inclusions larger than 21 μm in zinc melt, while the minimal detectable inclusion size in aluminum melt is slightly lower, at 18 μm. This discrepancy primarily stems from the analyzing system’s sensitivity to noise levels. Larger noise necessitates a higher noise threshold adjustment. This threshold mirrors the operational conditions at the testing site, establishing the minimum size of inclusions that the system can identify under such circumstances. Consequently, an elevated noise threshold results in an increased minimum detectable inclusion size. Variations between the testing environments for zinc and aluminum melts lead to differences in measurement noise, thereby affecting the lower limit of detectable inclusion size. During the tests, it was found that the small pores of the sampling tubes are more frequently blocked by inclusions in the zinc melt than in the aluminum melt, partly due to the high number of large-sized inclusions in the zinc melt, and partly due to the high slag thickness in the zinc melt, which lead to an increase in the viscosity of the zinc melt.

## 5. Conclusions

In this study, we developed an online analyzing system for assessing the cleanliness of liquid metal, utilizing the ESZ method to measure inclusion distribution. The system consists of pneumatic, embedded, and software modules. The pneumatic module incorporates a regulator with a negative feedback system to maintain output pressure close to the target. The embedded module adjusts signal gain and filter passband in real time and quantizes analog signals with high precision through optimal circuit design and parameter configuration. The host computer module manages data buffering, employs a custom data frame for transmission, and utilizes a finite state machine for data frame analysis to guarantee data transmission’s timeliness, completeness, and accuracy. It also implements a peak detection algorithm to extract voltage pulse and particulate matter information, displaying these data on the user interface.

Experimental results demonstrated the system’s ability to detect variations of the inclusions in the liquid gallium, showing a correlation between detected voltage pulse width and peak voltage pulse. The system’s efficacy in measuring inclusions in aluminum liquid at 800 °C and in zinc liquid was also validated.

Although the system can effectively monitor the size and numbers of inclusions within zinc melt in real time, enhancements in measurement continuity and stability are achievable. Continuous updates and improvements to each module can offer new perspectives and methodologies for online analyzing and quality control of zinc slag in the hot-dip galvanizing process.

## Data Availability

The data presented in this study are available from the corresponding author upon request. The data are not publicly available due to privacy restrictions.
